# Peer Review of “Cross-Modal Sensory Boosting to Improve High-Frequency Hearing Loss: Device Development and Validation”

**DOI:** 10.2196/55728

**Published:** 2024-02-09

**Authors:** ­ Anonymous

**Keywords:** audiology, hearing, high-frequency, wristband, develop, development, wearable, wearables, machine learning, phoneme, phonemes, hear, vibrotactile, vibration, vibrations, sound, sounds, hearing loss, loud noise, loud noises, noise pollution, hearing aids, hearing aid

*This is the peer-review report for “Cross-Modal Sensory Boosting to Improve High-Frequency Hearing Loss: Device Development and Validation”.*

## Round 1 Review

### Overall

- This study [1] reports on an interesting device with intriguing clinical implications for people with hearing loss.
- Innovative, and worthy of reporting on this technology, which could inspire other researchers
- But there are some issues that I feel require revisions:
  - Conflation of self-reported and objective benefit in the write-up
  - Lack of reporting the range and dispersion of the data—paper focuses on group means and gives very little ability to draw any inferences about individual participant variability
  - Lack of objective data about performance of the algorithm and participant performance for speech understanding
  - No data presented for the final questionnaire presented in the Methods
  - Presentation and discussion of results switches back and forth between benefit scores and raw scores in a way that is unclear and makes the paper difficult to follow and interpret at times
  - Some conclusions are presented without statistical results to support them
  - Some conclusions are stated too strongly given the sample size and study design
  - Lack of a limitations section to help reader contextualize the results

### Abstract

- “...improve their understanding of verbal communication.”: Please indicate that this is a self-reported or self-perceived understanding of verbal communication. I think it is important to distinguish the results from objective speech recognition testing (acknowledging that self-reported benefit is very important).
- “...greatest amount of benefit...”: Please indicate that it is a self-reported or self-perceived benefit.

### Introduction

Page 2: Authors indicate that auditory and vibrotactile information can be unconsciously and naturally integrated in the brain. It would be helpful if the authors could give some description/details of how the integration is hypothesized to occur—how long it takes and what neural/cognitive mechanisms might support it. Even if this is just a hypothesis, it would provide helpful context.

Page 3: “...can help individuals with high frequency hearing loss better understand speech communication...” Please indicate that it can help them self-report or self-perceive better speech communication.

Page 3: The last sentence is too strong. A device can help improve self-reported speech communication without translating to the types of benefits the authors describe in these various situations/environments. It could say something like “the evidence demonstrate the promise of this technology, which if further developed and refined holds promise for...”—something like this. I think it is OK to indicate that these kinds of benefits are possible in the future, but they are not directly supported by the results of this small study. Lots more work is needed.

### Methods

Not much detail about the machine learning algorithm is provided. More detail about how it filters background noise (BN) and identifies phonemes would be helpful. How was the algorithm trained? Assuming it was trained on speech, what regional accents were used?

Related to the above, it is not clear how the sham algorithm was used in developing the algorithm. Additional detail/description would be helpful.

Page 4: The authors mention that the algorithm performed poorly for some consonants that people with hearing loss have trouble hearing. It is not clear what level of performance constitutes poor performance and what level constitutes good performance for the phonemes that were selected for the algorithm. More context here would help the reader to understand the results. Understanding the algorithm’s accuracy is important for contextualizing the users’ results. It would be reasonable to suspect that the users’ results should be closely linked to the algorithm’s accuracy.

### Tasks

I am a bit confused as to why objective speech recognition testing was not completed. The self-reported benefit is absolutely important, but based on the Introduction, the reader is interested in knowing how objective speech recognition improved with the wristband for the selected consonants. If these data are available, it would be helpful to add them. If not, it would be helpful if the authors could explain—somewhere in the manuscript—why this testing was not completed/reported.

Final questionnaire: It does not seem like the results of the final questionnaire are reported in this manuscript. Given that the Abbreviated Profile of Hearing Aid Benefit (APHAB) is the only reported outcome measure, it would be helpful to add these results as well, as they represent something more holistic than the weekly APHAB results.

### Paradigm

Does the wristband provide any data logging to indicate how many hours per day the devices were worn? If not, this is not a major flaw but should be mentioned as a limitation because it seems like wear time could directly affect benefit.

#### APHAB

It might be helpful to the reader to clarify that higher raw APHAB scores indicate worse performance and lower scores indicate better performance but higher benefit scores represent more benefit or better outcomes.

### Participants

I would suggest adding the number who did and did not use hearing aids in this section. Any additional information regarding participants—gender, education, etc—would be helpful if it is available to report. Otherwise, I suggest adding that a limitation of the paper is the limited demographic information of the participants (combined with a small n), which makes it hard to determine if any participant-level characteristics might influence the benefit of the wristband.

The authors mention that if a clinical audiogram was unavailable, participants completed an audiogram via a mobile app. Then the authors provide an example, Mimi. Did everyone who used a mobile app use the Mimi app, or did some use other apps?

Relatedly, it would be helpful to report how many participants had clinical audiograms and how many used an app to provide context for the audiometric results.

### Results

One critique is that I did not feel like I got a very good handle on the descriptive statistics before the authors started showing group means (with SE of the mean) and comparisons (both over time and between subgroups of participants). I felt that the results emphasized group means (with SE of the mean), but I did not get a good sense of the range and dispersion of the data. In the Discussion, the authors start discussing the numbers of participants who started or ended at a specific APHAB overall score range, but I did not feel like I had the information in the paper to help me contextualize that discussion (because the results, as presented, do not give a very clear view of how individual participants may have performed).

To address the above point, I would strongly suggest adding a descriptive results table first that gives the means, maximums and minimums, and SDs for the overall APHAB scores and, possibly, APHAB benefit scores. It would be nice to see these values for the full participant group, as well as for the subgroups of participants with and without hearing aids (including the n in each group). It would be helpful to see the same data for the subscale scores (ease of communication [EOC], BN, reverberation) if it fits in the table, but I think the overall APHAB scores would be sufficient if space is an issue. Another consideration is that with only 16 participants, you could show the individual-level data for each participant, who would each be a row, and then give the group data in a different row. I defer to the authors on their preferences but would simply suggest that some revisions be made to give the reader a better grasp of the descriptive results.

In the section where subscale analyses are given, the write-up describes comparisons of subscale benefit scores between the different subgroups (with and without hearing aids) as well as comparisons, within a subgroup, of benefit scores to the baseline score. Throughout this section, it is hard to track which *P* values go with which comparisons. It is hard to read and interpret. Additionally, the information is presented slightly differently for each subscale, which makes it even harder to follow. Clearer written descriptions of each comparison being tested—then followed by the statistical numbers—would be beneficial for the reader. Additionally, using a parallel results presentation for each subscale would be helpful.

### Discussion

“...individuals with high frequency hearing loss are able to improve their understanding of speech communication...”: I would like to see it be specified that this is an improvement in self-reported or self-perceived understanding of speech communication. Previous hearing aid research shows there can be a placebo effect associated with the perception that one is wearing advanced technology [2].

“...participants were able to improve their ability to understand conversations during daily interactions.”: Same comment as above. Please indicate this is a self-reported or self-perceived ability to understand conversations.

“We further found that participants who started the study with a higher APHAB score experienced a greater improvement in their ability to understand speech by the end of the six week trial.”: As mentioned earlier in this review, this result is hard to interpret without any sense for the individual variability in the data. The results are presented as group means without clear maximums and minimums or SDs. Providing this information in the Results would help give context to this claim in the Discussion.

“Out of 16 participants, 14 ended the study with an APHAB score of 40 or below...”: This is again difficult to interpret without any sense for the individual-level data. At timepoint zero, the group mean is right around 40. It is not clear if ending the study at 40 or below indicates benefit or is just a reflection of peoples’ starting points. The discussion should be framed in terms of the amount of benefit people reported.

“Five participants started the study with an unaided APHAB of 50 points or higher...”: Again, a better sense of the individual-level data and dispersion would help give context for this. The Results are focused on means and then the Discussion brings up individual data, and it is hard to interpret the two together.

A small point but should this be <30 not >30 as written in the text?

“One potential hypothesis...”: It seems like this could also be due to having more room to improve their everyday speech understanding. I think it is important to acknowledge possible noncortical factors that could explain this finding (though I think it is fine to also leave the possibility that it reflects cortical characteristics).

“Participants without hearing aids benefitted the most...”: I think given the lack of statistical significance in the comparison of the group means, this needs to be toned down a bit. Perhaps something like “Participants without hearing aids demonstrated a trend toward higher self-reported benefit, though this did not reach statistical significance.” I know the authors reference the Cox 10-point criterion, but I am not sure that can be accurately applied to these data when the statistical test says the group means themselves are not statistically different (maybe related to the small sample size and variance in the data). Again, I would also like the benefit to be specified as self-reported or self-perceived.

“Given that this group started the study with a higher APHAB score...”: I did not find where there is a statistical test to justify this claim. This should be justified with a *t* test. Otherwise, I think it would be OK to specify that the *t* test did not show a statistical difference, but this group is trending toward having a higher baseline APHAB score.

“In this study, we demonstrated the addition of vibrotactile feedback in the presence of background noise enabled individuals who did not wear hearing aids to hear speech communication better...”: Again, would like to see it noted that this is a self-reported or self-perceived benefit.

The authors present the final average BN scores (eg, 28.95 and 40.04), but the section above seems to be focused on benefit scores. This reflects my earlier comment about providing more descriptive data upfront. It is hard to track how the authors switch between baseline and benefit scores, and without a descriptive table to refer to, it is difficult to contextualize some of the Discussion.

Related to the above, these scores are presented as being different but are they statistically different?

“...suggesting that those who use hearing aids may benefit from using vibrotactile feedback during conversations in background noise instead of using their hearing aids.”: I think this is much too strong of a conclusion for the data, study design, and sample size. This needs to be significantly toned down—as written, I think this is a reckless conclusion based on the limitations of the data. I could be OK with presenting this as an interesting finding worth future research to determine if the above could potentially be true. However, it would need to be framed by saying that this sort of clinical recommendation would require much larger, more rigorous studies with blinding of participants and researchers.

“Similar to our findings in background noise, we also found...”: From what I can see in the subscale results discussion, the difference between the group with and without hearing aids did not reach statistical significance. If this is true, it seems to be going too far to say that the wristband helped people without hearing aids the most. Here, I think it is OK to note that the results are trending in this direction as long as it is acknowledged that the results did not reach statistical significance.

“At the end of the trial, the group of participants who did not wear hearing aids showed an average reverberation score that was less than the average for the group who were regular hearing aid users.”: Was this tested statistically? From what I can tell, it looks like only the benefit scores are presented in the Results—not the raw scores. If the Discussion brings up the raw score (not the benefit), this should be presented in the Results section. Again, statistical results are needed to draw conclusions regarding the comparison of means.

“It is possible that individuals who use hearing aids may find haptic vibrations to be more helpful in reverberant environments...”: Similar to a comment above, I could be OK with presenting this as an area for future research, but I think it needs to be framed by noting the limitations of this study for drawing any clinical recommendations around hearing aids versus haptic vibration.

“Upon completion of the trial, the average EOC score...”: Similar to previous comments, it seems that the Results only present benefit scores but now the Discussion mentions raw EOC scores for the group with and without hearing aids. If raw scores are mentioned in the Discussion, they should be presented in the Results.

This section ends by noting equivalent ending EOC scores for the group with and without hearing aids; a statistical result should be presented to make this claim (and should be presented in the Results section).

One additional note: Results from the final questionnaire do not seem to be presented. Is there a reason for this? Given that the APHAB is the only outcome measure, it would be beneficial to see results from the final questionnaire in this paper alongside the APHAB. The final questionnaire also measures something a little different than the APHAB—it is more holistic for the whole field trial experience.

### Conclusion

Same comments as before about noting that this study applies to self-perceived or self-reported benefit.

“We found that vibrotactile feedback provides more benefit for those without hearing aids than for those with hearing aids...”: From what I see in the Results section, the statistical results do not support this conclusion. The 10-point criterion from Cox cannot be applied if we are not sure the group means themselves are even different (as indicated by the insignificant *P* value). I think it is OK to say the data are trending in this direction and that the small n may render the study underpowered to detect this difference at *P*<.05. Future work is needed to establish whether this claim is true. For now, I would argue it needs to be softened based on the findings and limitations of the study design.

Finally, I suggest adding a limitations section, which could note limitations around:

- Small n
- Reliance on self-report data without objective speech-testing data
- Potential for placebo effect to influence results
- Small n makes it difficult to discern whether/how individual and demographic characteristics could affect ability to integrate the haptic vibrations and benefit from the wristband—some characteristics one might wonder about include baseline cognitive ability, education level, differences in underlying degree/configuration of hearing loss, or duration of hearing loss
- Use of nonclinical audiogram for some participants (a minor limitation but should be noted)
- No information on how many hours per day the wristband was worn. One might hypothesize that outcomes could be related to wear time. Furthermore—beyond raw wear time—we also do not have information about the richness/complexity of auditory information processed through the wristband

## Round 2 Review

I appreciate the authors’ thorough revision in response to reviewer feedback, and I found this version to be very much improved. It has been a pleasure reviewing this paper and learning more about the authors’ interesting work on this novel device, which is now more clearly and thoroughly explained in this newest version of the paper.

I have only a few suggested minor revisions remaining, as follows:

- In the Results section of the Abstract, it says “those without hearing aids showed a 10.78 point greater drop in average APHAB benefit score at 6 weeks.” I believe this should read 10.78 higher APHAB benefit score. It would be a drop in score from baseline to the 6-week score if discussing the global APHAB score, but if discussing the benefit score, then the score increased from baseline to 6 weeks.
- In the Results section of the Abstract, most of the results are discussed as the group average, with only one result framed in terms of non–hearing aid users versus hearing aid users. It might be helpful to more clearly specify that when the average results are presented—it is across all participants. I do not have a strong preference on this, just something I noticed.
- In the last paragraph of the Introduction, it says “...can help individuals with high frequency hearing loss to feel more confident in their ability to understand speech communication.” Although I understand why the authors are making this inference from the APHAB, it does not feel quite supported enough to jump from the APHAB results to a statement about participants’ confidence. I would strongly suggest editing this to be in line with the language used throughout the rest of the paper (eg, increasing subjective assessment of speech ability, increasing self-rated communication ability, or decreasing self-perceived hearing difficulty in daily communications).
- At the end of the APHAB section under Tasks, where it says “Higher benefit scores indicate...,” I would also suggest adding the calculation for the benefit score as unaided – aided; then, it could be deleted from the next section.
- In Table 1, I would suggest adding a column to indicate which participants had a professional hearing test and which used the app option.
- For the Table 2 legend, I would suggest specifying how precision and recall are calculated in terms of true positives, false positives, etc. Additionally, it would be helpful to know how the *F*_1_-score is calculated.
- In the Results section comparing non–hearing aid users to hearing aid users, the sentence about the 10.78-point difference could be made clearer if it specified that the non–hearing aid users had a 10.78-point higher benefit score than the hearing aid users (rather than just saying there is a difference).
- In the same section of the Results, it says “...average APHAB benefit over baseline...”—since the benefit score reflects a reduction in the APHAB score, I would suggest framing benefit not as being “over baseline” but rather “from baseline.”
- In the Discussion section, where it says “Out of 16 participants, 14 ended the study with an APHAB score of 40 or below....” I think this would be more helpful if it said how many of them started the study with a score of 40 or below. I do not have a strong preference, however. Now that individual data are presented, it is much easier to contextualize the results.
- In the Discussion section, it says “It is also possible that participants who started the study with a lower APHAB score had more room for improvement.” I think this should say a higher APHAB score, as higher scores mean more perceived difficulty.
- In the Conclusion, it mentions that the study was underpowered to detect the difference between hearing aid users and non–hearing aid users at *P*<.05. This is presented for the first time in the Conclusion, which seems out of place. I would suggest first mentioning this in the Limitations section above. It could also be mentioned in the Conclusion, though, because it’s an important point—but reading new information in the conclusion was a bit jarring.

### Very Minor Comments

- First paragraph under APHAB under Tasks, suggest revising “they are asking” to “they ask”
- In the same section, suggest revising the two instances of “was referring” to “referred”
- For the Results section that discusses the BN score, it should read “16.99 points higher than those with hearing aids” (“with” is missing)

## Abbreviations

APHAB: Abbreviated Profile of Hearing Aid Benefit
BN: background noise
EOC: ease of communication
